# Influence of dental implant surfaces on oral biofilms and host immune response

**DOI:** 10.1080/20002297.2025.2607199

**Published:** 2025-12-25

**Authors:** Jon J. Vernon, El Mostafa Raïf, Jensen Aw, Ed Attenborough, Animesh Jha, Thuy Do

**Affiliations:** a Division of Oral Biology, School of Dentistry, University of Leeds, Leeds, UK; b Attenborough Dental Laboratories Ltd., Nottingham, UK; c School of Chemical and Process Engineering, University of Leeds, Leeds, UK

**Keywords:** Oral microbiology, biofilm, material science, antimicrobial, peri-implantitis, infection, immune response

## Abstract

**Background:**

Peri-implantitis, driven by microbial‒host immune interactions, is the leading reason that dental implants fail. Implant surface design plays a crucial role in microbial colonization.

**Objective:**

To investigate how surface characteristics of implant materials impact periodontal disease biofilm formation and host immune response.

**Design:**

Biofilms, cultured on Ti-6Al-4V and CoCr disks, had biomass quantified by crystal violet and microbial populations by agar enumeration. We assessed the influence of Ti-6Al-4V post-processing treatments on surface chemistry (energy dispersive spectroscopy), topography (optical profilometry) and microbial dynamics (through complex oral biofilm culture and 16S rRNA sequencing). To evaluate immune responses, biofilms were co-cultured with dysplastic oral keratinocytes, and IL-6, IL-8, IL-1β, TNFα and GRO-*α* ELISAs were performed.

**Results:**

Sandblasting markedly increased surface roughness (3.9 vs 0.2–0.6 R_a_), biomass (0.72–0.99 vs 0.13–0.62 AU) and total viable counts (TVC). Ti-6Al-4V demonstrated significant enrichment of firmicutes compared to CoCr, together with increased proportions of sulphate-reducing and periodontal disease-associated taxa. Rougher surfaces provoked stronger immune activation under microbial challenge, highlighting the link between topography and host response.

**Conclusions:**

Surface roughness influenced biofilm formation and inflammation. Assessment of implant materials should integrate microbial and cellular responses for deeper insights. Smoother surfaces, combined with antimicrobial coatings may help reduce peri-implant disease.

## Article highlights

To gain a greater understanding of new implant materials or coatings, it is important to assess them in combination with microbial burdens and the host cellular environment.

Higher surface roughness of implants supports increased microbial loads, which subsequently induces a greater epithelial cell immune response, characterized by increased expression of pro-inflammatory cytokines.

To counteract peri-implant disease reducing the microbial load through the production of smoother surfaces is an important building block to combine with alternative approaches, such as antimicrobial coatings or drug delivery systems.

## Introduction

Dental implant placements are increasing annually, with projections indicating this trend will continue [1]. However, with peri-implantitis affecting approximately 20% of cases [2] and failure rates estimated between 5 and 10% [3–7], the need for improved implant surfaces is evident. The recently proposed biofilm-mediated inflammation and bone dysregulation (BIND) hypothesis suggests that peri-implantitis pathogenesis arises from complex interactions between microbial biofilms, the host immune system and bone cells, promoting disease progression and contributing to shifting microbial populations [8]. Therefore, it is of utmost importance to assess the efficacy of implant materials not only in terms of microbial colonisation but also in the context of their interface with host immunity and tissue responses.

Considering the limitations of animal models, such as anatomical differences, varied healing responses and financial/ethical challenges [9]*, in vitro* approaches are crucial for evaluating novel implant surfaces. These methods allow for enhanced control over experimental conditions and are valuable for generating detailed material, chemical and biological data.

Since peri-implantitis is primarily an immune-mediated disease [10], analysing microbial load alone is insufficient, doing so neglects the central role of dysregulated inflammation. Host immune response, particularly cytokine expression, offers critical insights into material biocompatibility and disease potential. Indeed, early increases in TNFα within healthy gingival crevicular fluid may signal the onset of inflammatory cascades and tissue remodelling [11].

In this study, we combined biological and physical techniques to characterise dental implant materials and surface finishes for their potential to exacerbate or mitigate peri-implantitis-associated factors. Using a newly developed co-culture model with oral epithelial cells, we evaluated post-processing techniques to identify favourable surfaces for *in vivo* application.

## Materials and methods

### Implant material manufacture

Test materials were manufactured at Attenborough Dental Laboratories Ltd. Disks of cobalt-chrome (CoCr) and grade five titanium alloy (Ti-6Al-4V) (7 mm ø, 2 mm height) were produced via direct metal laser sintering (DMLS) using an EOS M270 (EOS, Germany). Post-processing generated three surface finishes; Al_2_0_3_ sandblasted (particle size 50 µm), high polish (by bench disk sander and rubber wheel) and electrolytic polish (30 min). For etched surfaces, high-polished Ti-6Al-4V disks were submerged in either 37% HCl, 98% H_2_SO_4_ or 69% HNO_3_ for 24 h at room temperature. All materials were ultrasonically cleaned (49 kHz; 5 min in 70% ethanol, 10 min in dH_2_O), autoclaved (121 °C for 15 min) and oven dried (40 °C for 1 h) before use.

### Surface roughness determination

Surface topography was assessed using non-contact optical profilometry (Proscan 2200, Scantron Ltd., UK). Mean roughness values from three measurements across three replicates were reported separately for the X- and Y-axes.

### Five-species *in vitro* oral biofilm model

#### Bacterial strains and growth conditions

Five-species biofilms were composed of *Streptococcus salivarius* (ATCC 7073), *Actinomyces naeslundii* (ATCC 27038), *Fusobacterium nucleatum* (ATCC 10953), *Prevotella intermedia* (OMZ 248) and *Porphyromonas gingivalis* W83 (ATCC BAA-308). All strains were initially cultured on blood agar (Oxoid, UK) supplemented with 5% horse blood (E&O Laboratories, UK) and brain heart infusion (BHI) broth (Sigma, UK). *S. salivarius* and *A. naeslundii* were incubated at 37 °C in 10% CO_2_ for 48 h, while *F. nucleatum*, *P. intermedia* and *P. gingivalis* were incubated anaerobically (10% H_2_, 10% CO_2,_ 80% N_2_) in a Whitley M55 Workstation (Don Whitley Scientific, UK) for 48–96 h, depending on species.

### Experimental design and model configuration

Oral biofilms were established on implant materials using a modified version of a validated model [12,13]. Disks of implant materials replaced hydroxyapatite-coated pegs to simulate the peri-implant environment. Hydroxyapatite disks served as substrata for positive growth controls, while negative controls were Ti-6Al-4V disks treated with 1% Virkon solution (200 µL) prior to inoculation.

Implant disks of various materials and surface finishes (Supplementary Table 1) were coated with sterilised human saliva (500 µL; supplementary materials) via incubation in 48-well plates for 5 h at 37 °C with 65 rpm shaking. Disks were transferred to fresh 48-well plates for bacterial inoculation. Overnight BHI cultures of *S. salivarius* and *A. naeslundii* were diluted to OD_600_ 0.2 and added to pre-warmed artificial saliva medium (4:1 artificial saliva (supplementary material): heat-inactivated human serum), with inocula of 1 × 10^5^ and 2 × 10^5^ colony-forming units (CFU), respectively. Disks were incubated anaerobically overnight.

Anaerobic species (*F. nucleatum,* 6 × 10^6^, *P. gingivalis,* 6 × 10^7^ and *P. intermedia,* 6 × 10^6^ CFU mL^−1^) were inoculated into artificial saliva, prior to disk addition. After 24 h, disks were transferred to a new 48-well plate for a second anaerobe inoculation. Disks were transferred daily into fresh sterile artificial saliva medium for eight days. On day 11, biofilms were washed three times with PBS and either transferred to fresh plates for biomass quantification (crystal violet assay) or into tubes with 500 µL pre-reduced PBS and vortexed (1 min) for agar enumeration. All implant materials were tested in both technical and biological triplicate.

### Viable counts

Homogenised biofilms were serially diluted up to 10^−7^ in PBS and plated on blood agar (incubated at 10% CO_2_, 37 °C for 48 h) for *S. salivarius* and *A. naeslundii* enumeration, and blood agar-supplemented with 75  mg L^−1^ vancomycin (incubated anaerobically, 37 °C for 5 days) for *P. intermedia*, *P. gingivalis* and *F. nucleatum*. Colonies were enumerated based on morphology and pigmentation compared to pure culture (Supplementary Figure 1) as per Naginyte et al. [13].

### Crystal violet biofilm mass determination

Biofilm biomass was quantified using the crystal violet assay described by O'Toole (2011) [14]. Briefly, biofilm-coated disks were washed twice with PBS and then incubated in 1 mL of 0.1% crystal violet for 15 min at ambient temperature. The dishes were rinsed with tap water four times and air-dried overnight. Stained biofilms were solubilized in 1 mL of 30% acetic acid for 15 min, and the absorbance of the resulting solution was measured at 550 nm, using 30% acetic acid as the control. Duplicate wells were used per sample, and background absorbance from control disks was subtracted to correct for non-specific dye retention.

### Complex microbiota implant biofilm model

To better replicate *in vivo* interactions, complex oral biofilms were cultured on implant materials to assess microbial shifts across different surfaces. Oral microbiota samples were collected with ethical approval from the University of Leeds Dental Research Ethics Committee (Ref: 111021/TD/334). Saliva, plaque (from natural teeth) and tongue scrapings from three healthy subjects were pooled under anaerobic conditions to make a single sample used as the inoculum for all complex oral community biofilm formations on implant disks. Biofilms were cultured in artificial saliva medium, refreshed twice weekly for 21 days, then harvested for molecular analysis.

### 16S rRNA gene sequencing

DNA from implant disk-derived biofilms was extracted using a QIAamp PowerFecal Pro DNA Kit (Qiagen Ltd., UK) and quantified using a Quant-iT™ PicoGreen® dsDNA Kit (Invitrogen, UK). 16S rRNA gene sequencing (V3–V4 region) was performed by Novogene (UK) Company Ltd., using Illumina chemistry. Bioinformatic analysis included quality control, taxonomic annotation, diversity and functional analyses using Qiime2 [15] and PICRUSt2 [16].

### Scanning electron microscopy

Biofilms on disk were washed with PBS and fixed with 2.5% (v/v) glutaraldehyde at room temperature for 3 h. After three PBS washes, samples were dehydrated in ethanol (30%, 60%, 80%, 97% and (3×) 100%) for 15 min each, air-dried, and sputter coated with gold (Agar Auto Sputter Coater, Agar Scientific Ltd., UK). SEM imaging was performed using a Hitachi S-3400N (Hitachi Science and Technology, Japan).

Elemental mapping of implant materials prior to biofilm formation was conducted via energy dispersive spectrometry (EDS), with a 2 × 60 mm^2^ Flash6 (Bruker, Germany). Images were captured at 500× magnification at 20 kV in triplicate, with the mean values reported.

### Co-culture with epithelial monolayers

**Figure 1.**
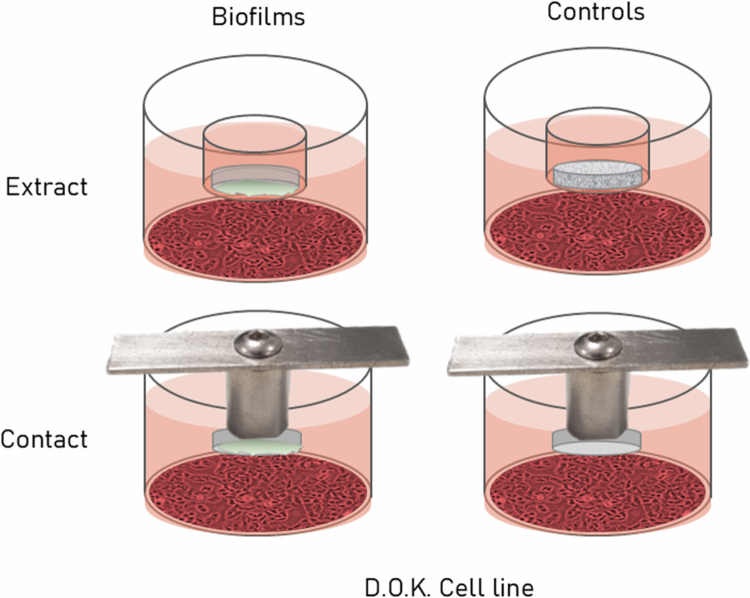
Implant disk-derived biofilm co-culture configuration. Dysplastic oral keratinocyte (D.O.K.) layers were exposed to either biofilms placed in tissue culture inserts (0.4 μm) positioned above the cells (top, extract assays) or suspended in the shared media via attachment to a sterile, custom titanium device with biologically inert steri-strip adhesive tape (bottom, contact assays).

Complex biofilms seeded from the combined oral samples of three healthy volunteers were used to assess the immune marker response in all co-culture assays. Dysplastic oral keratinocyte (D.O.K.) monolayers were established in 12-well plates by seeding 1 × 10^5^ cells/well in DMEM supplemented with 2 mM glutamine, 5 µg mL^−1^ hydrocortisone and 10% foetal bovine serum (FBS); (D.O.K. media). After 24 h incubation (37 °C, 5% CO_2_), monolayers were washed with PBS and replenished with fresh media. Mature biofilms were co-cultured with D.O.K. monolayers using contact and extract assays. In extract assays, biofilms were placed in tissue culture inserts (0.4 μm) enabling soluble factors to diffuse. For contact assays, implant biofilms were attached to custom titanium devices using biologically inert steri-strips (3 M) and suspended in shared growth media, enabling direct interaction with epithelial cells; (Figure 1). After 24 h, supernatants were collected for cytokine quantification (IL-8, IL-6, IL-1β, TNFα and GRO-*α*) via DuoSet ELISA (Biotechne, USA), with concentrations normalised to unstimulated monolayer controls. Cell viability was assessed, post-washing with 1 mL sterile PBS to ensure only D.O.K. cells contributed to colorimetric response, using an MTT-based *in vitro* toxicology assay kit (Sigma, USA). All assays were performed in triplicate, with acellular wells as controls.

### Statistical analyses

Statistical analyses were performed using IBM SPSS Statistics version 29.0.0. Groups were compared using ANOVA with Tukey multiple comparison of means. The level of significance was set at *p* < 0.05.

## Results

### Implant material properties

**Figure 2.**
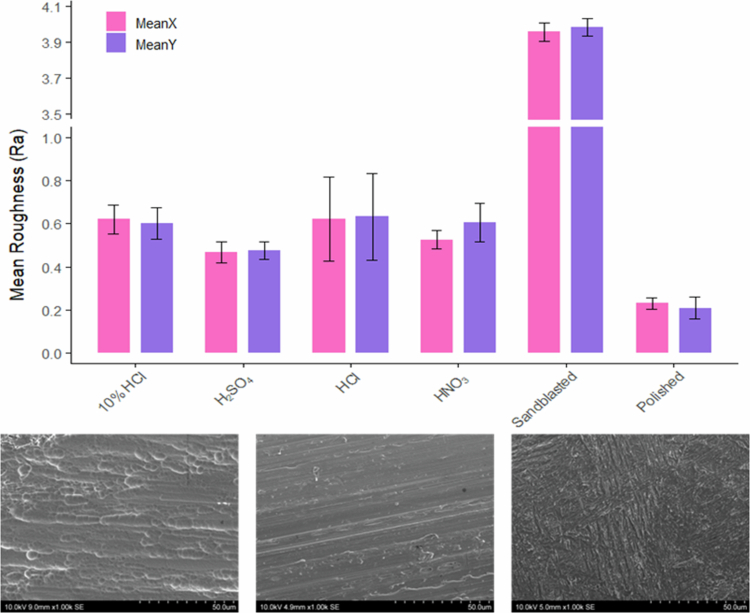
Acid-etched titanium material assessment. Top: Mean roughness (±standard error of the mean) measured by optical profilometry. Bottom: Scanning electron microscopy images of acid-etched titanium surfaces at 1,000× magnification (10 kV) (left – sulphuric acid-etched, middle – nitric acid-etched, right – HCl-etched) demonstrate clear visual differences.

Optical profilometry demonstrated that different post-processing methods produced a range of surface roughness values (0.209–3.983 R_a_) and distinct topographical patterns; (Figure 2). The high-polished finish showed the lowest roughness (x = 0.231, y = 0.209 R_a_), while sandblasted Ti-6Al-4V exhibited the highest (x = 3.958, y = 3.983 R_a_). Among the etched samples, sulphuric acid produced the smoothest surface (x = 0.468, y = 0.475 R_a_), characterised by distinct circular etching patterns; (Figure 2), whereas HCl resulted in rougher surfaces (x = 0.621, y = 0.633 R_a_) with deep, intersecting grooves.

EDS confirmed the predominance of core elements; Ti (83.26–86.28%), Al (5.27–5.50) and V (3.24–4.05). Trace elements were also detected, chlorine (0.0001%) on HCl-etched surfaces and sulphur (1.99%) on sulphuric acid-etched surfaces (Supplementary Table 1). Sandblasted Ti-6Al-4V resulted in distinct areas of co-located aluminium (green) and oxygen (yellow) deposits (Supplementary Figure 2).

### Five-species *in vitro* oral biofilm biomass

**Figure 3.**
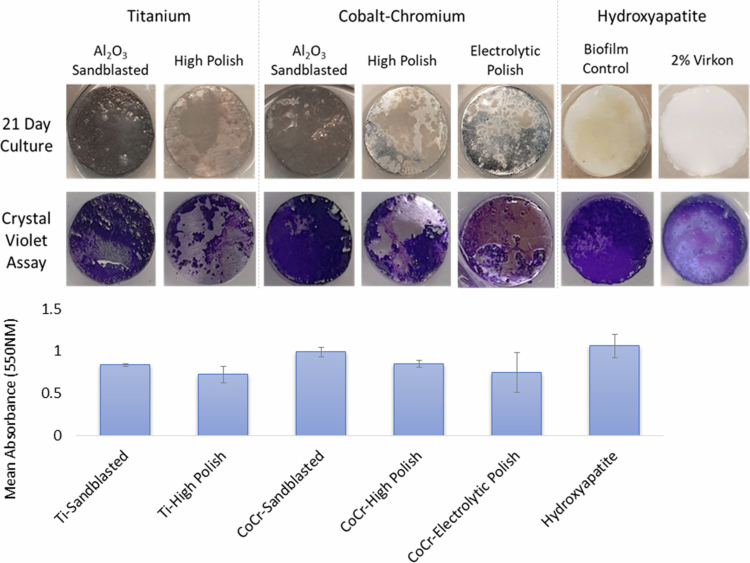
Top - Representative images of complex oral biofilms after 21-day culture, with and without crystal violet staining. Bottom – Mean biofilm biomass (±standard error of the mean) from Ti-6Al-4V, CoCr and control surfaces, determined by crystal violet assay at 550 nm. Standard error of the mean is shown.

Sandblasted surfaces of both Ti-6Al-4V and CoCr showed the highest levels of biofilm accumulation (0.84 ± 0.01 and 0.99 ± 0.06 AU, respectively), comparable to the hydroxyapatite control (1.06 ± 0.14 AU); (Figure 3). In contrast, high-polished finishes reduced biomass on both Ti-6Al-4V (0.72 ± 0.09 AU) and CoCr (0.85 ± 0.04 AU) compared to their sandblasted counterparts. Overall, Ti-6Al-4V implant materials supported lower biofilm accumulation than CoCr disks.

### Five-species biofilm population enumeration

**Figure 4.**
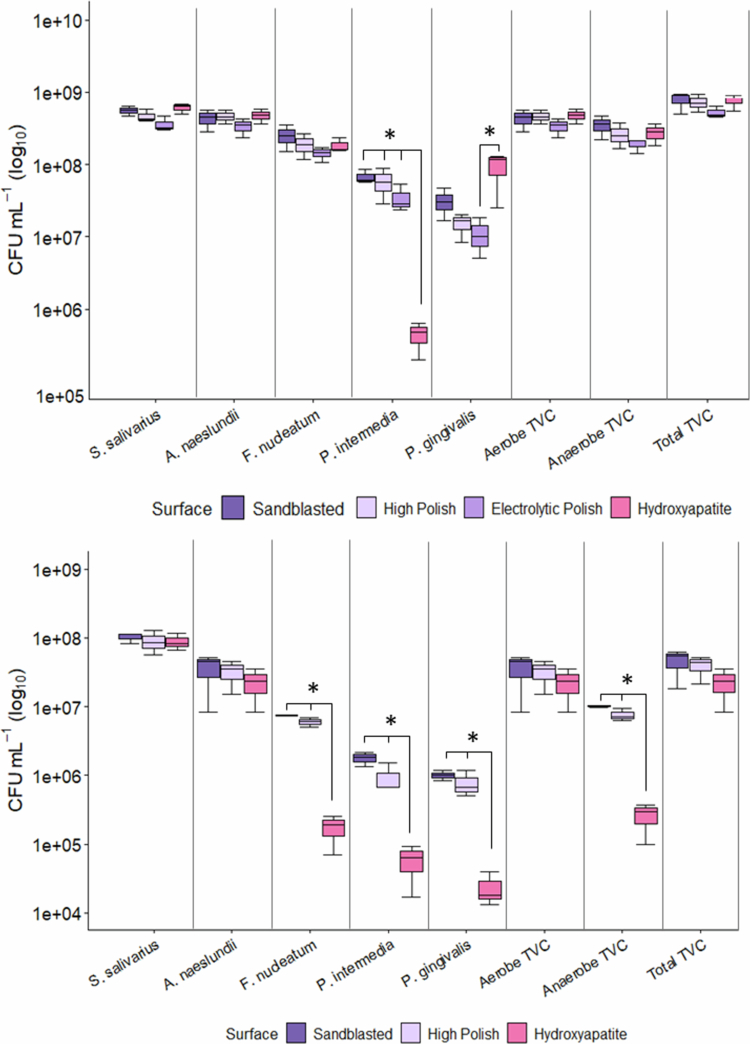
Boxplots showing colony-forming unit (CFU) counts (log₁₀ scale) for mature five-species oral biofilms grown on different surface finishes of CoCr (top) and Ti-6Al-4V (bottom). Data are presented for individual species and grouped as aerobes, anaerobes and total viable counts (TVC). Boxes represent the interquartile range (IQR), horizontal lines indicate medians and whiskers show the range within 1.5 × IQR. Significant differences (*) were observed (*p* < 0.05), based on ANOVA with Tukey HSD.

TVCs were approximately 1 log_10_ lower in experiments testing Ti-6Al-4V (~1.39 × 10^8^ ± 1.15 × 10^7^ CFU mL^−1^) compared to CoCr (~1.27 × 10^9^ ± 1.28 × 10^8^ CFU mL^−1^); (Figure 4). However, in each experiment, the hydroxyapatite controls showed similar bacterial counts to the implant materials; CoCr: 1.37 × 10^9^ ± 1.03 × 10^8^ and Ti-6Al-4V: 1.11 × 10^8^ ± 7.80 × 10^6^ CFU mL^−1^. *P. gingivalis* was notably enriched on hydroxyapatite (9.06 × 10^7^ ± 3.30 × 10^7^ CFU mL^−1^) compared to CoCr (~2.31 × 10^7^ ± 1.21 × 10^7^ CFU mL^−1^), while *P. intermedia* counts were over 2 log_10_ lower (4.39 × 10^5^ ± 1.27 × 10^5^ vs 6.28 × 10^7^ ± 1.30 × 10^7^ CFU mL^−1^). On Ti-6Al-4V disks, total anaerobe populations were markedly higher than on hydroxyapatite; 1.79 × 10^7^ ± 5.74 × 10^5^ and 2.54 × 10^5^ ± 7.98 × 10^4^ CFU mL^−1^, with this trend consistent across all three anaerobic species; (Figure 4).

### Complex biofilm population analysis by 16S rRNA sequencing

**Figure 5.**
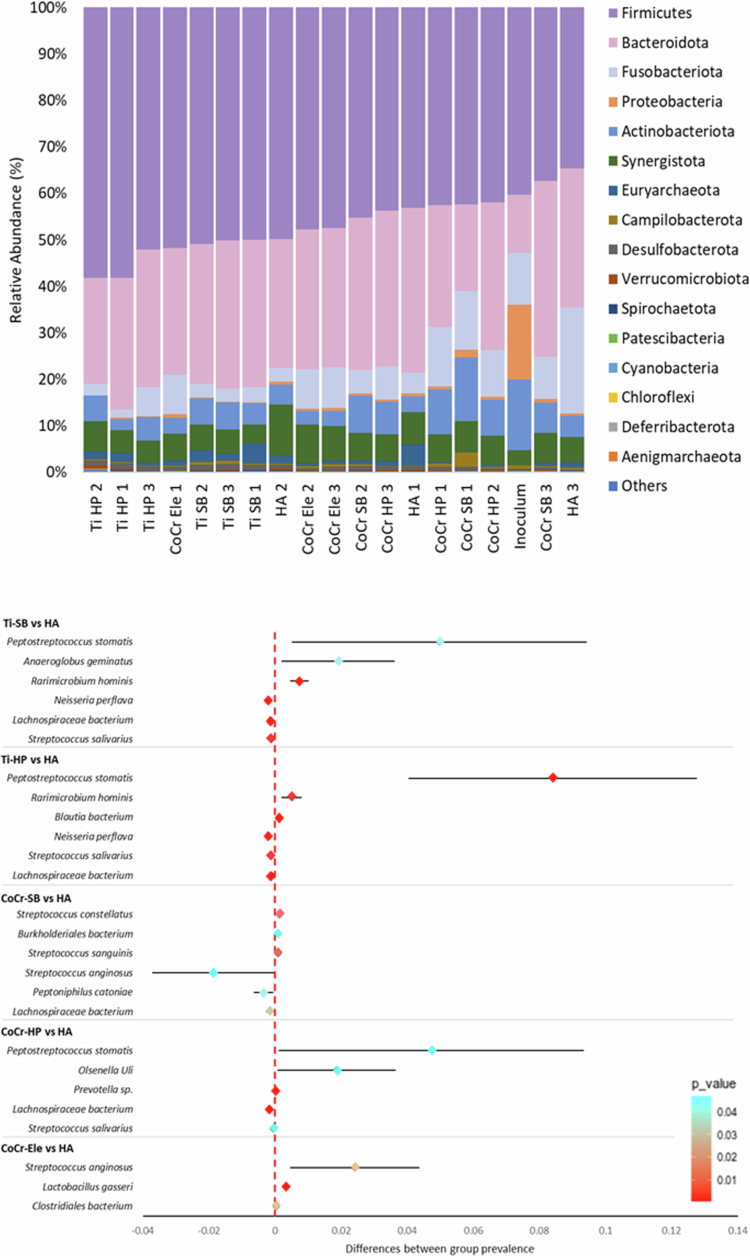
Microbial relative abundance of complex oral biofilms on different implant surfaces by 16S rRNA sequencing. Top: Relative phyla abundance on polished (HP) and sandblasted (SB) titanium, cobalt-chromium and hydroxyapatite disks. The inoculum sample refers to the pooled material from all three donors. Bottom: Forest plot displaying the significant differences between groups of test materials at a species level. T-test significance is shown, with confidence intervals represented by horizontal lines.

For complex oral biofilm cultures on implant materials, Firmicutes were enriched on Ti-6Al-4V compared to CoCr and hydroxyapatite (Figure 5). Within each material type, highly polished surfaces supported higher Firmicutes proportions than sandblasted finishes; (Ti-6Al-4V (56.1 vs 50.3%) and CoCr (42.7 vs 41.6%)). Desulfobacterota were significantly enriched on Ti-6Al-4V (1.2%) vs hydroxyapatite (0.7%), with significant beta diversity (*p* = 0.01) for sandblasted and high-polished Ti-6Al-4V.

At the species level, biofilms were dominated by the same top 20 taxa, including *P. gingivalis*, *Peptostreptococcus stomatis* and *Fusobacterium spp.* Significant increases in *P. stomatis* (*p* = 0.046) and *Olsenella Uli* (*p* = 0.045) were observed on CoCr compared to hydroxyapatite. On Ti-6Al-4V, *P. stomatis* (*p* = 0.006), *Anaeroglobus geminatus* (*p* = 0.037) and *Rarimicrobium hominis* (*p* = 0.009) were significantly elevated relative to hydroxyapatite (Figure 5). Additionally, *Treponema socranskii* was significantly more abundant on Ti-6Al-4V than CoCr; (*p* = 0.013).

Functional analysis revealed clear material-specific differences, with Ti-6Al-4V and CoCr forming distinct clusters; (Supplementary Figure 3). Clusters of genes (COGs) enriched on implant materials vs hydroxyapatite were primarily involved in transcription regulation, cell structure biogenesis and carbohydrate metabolism/transport. In contrast, genes related to lipid/lipoprotein metabolism were depleted.

### Complex oral biofilm biomass

Crystal violet assay indicated that biofilm quantification generally correlated with surface roughness (Figure 2 and Supplementary Figure 4). Sandblasted disks, which exhibited the highest roughness, supported the greatest biofilm accumulation (0.78 ± 0.05 AU). Among acid-etched surfaces, biomass levels also reflected roughness: HCL, nitric acid and sulphuric acid-etched Ti-6Al-4V showed mean absorbances of 0.62 (±0.06), 0.61 (±0.07) and 0.13 (±0.04) AU, respectively.

### Co-culture modelling

### Cell viability impact

A statistically significant relationships between biofilm biomass and remaining D.O.K. cell viability post-co-culture was identified using Spearman's rank correlation (*p* = 0.0498). After 24 h of co-culture with implant disk-derived biofilms, D.O.K. cell viability was substantially lower in contact assays (6–21%) compared to extract assays (28–84%); (Supplementary Figure 5). Sulphuric acid-etched surfaces, which exhibited the lowest biofilm biomass, retained the highest cell viability (21%).

### Immune response

**Figure 6.**
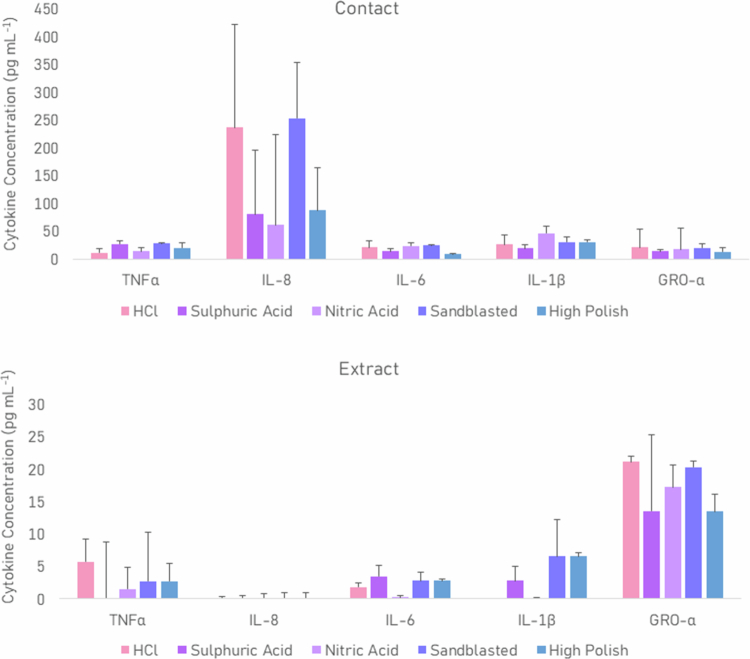
Mean (±standard error of the mean) immune response of D.O.K. cell monolayers to complex biofilms cultured from combined oral samples of three healthy volunteers on acid-etched titanium implant surfaces, both direct contact (top) and 0.22 µm tissue culture insert filtered extracts (bottom). Concentrations were normalised by subtracting data from unstimulated cell monolayers.

Cytokine concentrations were vastly higher in contact experiments (8.8–253.4 pg mL^−1^) compared to extract assays (0.0–21.2 pg mL^−1^), (Figure 6). In contact assays, sandblasted disks generally elicited the strongest immune responses, with TNFα, IL-8, IL-6 IL-1β and GRO-*α* concentrations of 27.7 (±2.3), 253.4 (±100.5), 25.4 (±0.4), 30.3 (±10.1) and 181.0 (±7.8) pg mL^−1^, respectively. In contrast, sulphuric acid-etched disks induced the lowest cytokine responses among acid-etched surfaces, particularly for IL-6 (14.4 ± 4.0 pg mL^−1^), IL-1β (19.7 ± 5.9 pg mL^−1^) and GRO-*α* (19.1 ± 3.4 pg mL^−1^). No statistical significance was observed.

## Discussion

This study aimed to assess the influence of different implant materials and surface finishes on oral biofilm accumulation and its impact on host immune responses. By understanding these combined factors, we sought to gain a greater understanding into the optimal implant surfaces for the prevention of peri-implant disease.

Initially, we sought to adapt an existing *in vitro* periodontal biofilm model to embed a variety of implant materials. The five-species, serum-rich model from Naginyte et al. [12], was validated on hydroxyapatite pegs, so this is its first application to dental implant material testing. The similarity in microbial composition and biomass observed between implant materials and hydroxyapatite confirms the model's translatability. When assessing the core materials, Ti-6Al-4V surfaces exhibited slightly lower biomass than CoCr, a trend mirrored in TVCs. However, all three anaerobic species were more abundant on Ti-6Al-4V than hydroxyapatite, suggesting titanium may favour anaerobic colonisation, potentially due to its surface physico-chemical properties or selective protein adsorption [17].

When comparing the microbiological and topography data of the different surface treatments, the results aligned with prior research linking surface roughness to higher biomass [18–21]. Our roughness values (~0.2 for polished; >1.3 R_a_ for sandblasted) were consistent with the literature [18,20,22]. Interestingly, a systematic review of 62 clinical studies reported the highest failure rates in rough-surfaced implants, notably with sandblasted materials (5.16%) [23]. Similarly, Albouy et al., observed greater peri-implantitis severity and disease progression in roughened implants in canine trials [24]. Since our data suggests that these surfaces correlate with greater accumulation of microbial biofilms, it supports the notion of the material-microbe-immune response relationship as crucial to disease causation.

SEM imaging supported this relationship, demonstrating patchy biofilm with dense peaks on polished surfaces (Supplementary Figure 6), while sandblasted materials harboured more complex, topography-penetrating biofilms, consistent with prior work [18]. However, the relationship between surface roughness and biofilm formation is not always straight forward. Schmidt and colleagues found no microbial load differences across implants receiving varying levels of instrumentation [25]. Some studies suggest a roughness threshold around 0.2 R_a_, beyond which little benefit would be achieved. Bollen et al supported this notion, reporting minimal differences in bacterial populations between machined Ti abutments (0.2 R_a_) and highly polished ceramics (0.06 R_a_) [22].

Furthermore, the translation of roughness parameters to clinical scenarios is not always clear cut. A longitudinal clinical trial found that although dual-etched surfaces increased plaque indices, this did not correlate with clinical markers for peri-implantitis, such as bone resorption [21]. Similarly, one study indicated that while sandblasted surfaces (>2.0 R_a_) harboured more bacteria *in vitro*, these differences were not observed *in vivo* using intra-oral devices [26]. This discrepancy may stem from the use of BHI broth *in vitro*, whereas the serum-based medium used here may more closely mimic oral conditions.

Notably, de Avila et al. observed a 6.1-fold reduction in biomass on titanium versus zirconia, despite matched surface roughness, suggesting chemical composition plays a key role in bacterial adhesion, potentially due to variations in surface free energy and hydrophobicity [27]. Our EDS analysis revealed that post-processing altered surface composition; sandblasted Ti-6Al-4V retained Al_2_0_3_ particles, evidenced by localised patches of aluminium and oxygen even after washing and sonication; (Supplementary Figure 2), while sulphuric acid-etched surfaces contained traces of residual sulphur (Supplementary Table 2). These chemical residues may have antimicrobial or even stimulatory effects and contribute to altered biomass [28]. Nonetheless, since dental implants rapidly acquire a salivary pellicle *in vivo*, interactions with salivary proteins may ultimately have a more decisive role in microbial colonisation [29].

While the five-species model captures key periodontal pathogens, peri-implantitis modelling may benefit from additional species specific to the disease [30], as emerging evidence suggests distinct population differences between periodontitis and peri-implantitis [31]. To reflect this, we used a complex oral inocula, allowing selective enrichment of species suited to the implant environment and better mimicking *in vivo* conditions.

16S rRNA sequencing revealed that Desulfobacterota were enriched in Ti-6Al-4V compared to hydroxyapatite. These sulphur-reducing bacteria generate hydrogen sulphide via the sulphite reduction pathway [32,33], which can degrade the protective TiO_2_ layer, resulting in corrosion, fatigue and fractures [34]. Additionally, peri-implantitis-associated species such as *P. stomatis* and *A. geminatus* [35,36] were significantly more abundant on Ti-6Al-4V. *A. geminatus* has been linked to elevated *P. intermedia* levels and upregulation of proteolytic pathways, potentially promoting bone degradation [37]. Network analysis further associated *A. germinatus* with increased caspase-1 expression in the epithelial tissues [38], promoting IL-1β activation and amplifying inflammatory responses [39].

*T. socranskii,* a red complex periodontal pathogen, was more abundant on Ti-6Al-4V than CoCr. This may relate to Ti ion release, which has been shown to favour anaerobic bacteria [40]. A potential feedback loop could exist, where biofilms accelerate titanium corrosion, increasing ion release, which further promotes pathogenic biofilm formation. While mechanisms remain unclear, one hypothesis suggests Ti ions interact with the TiO_2_ layer, reducing oxygen availability and creating conditions favourable to anaerobes. Once the protective TiO_2_ layer is compromised, corrosion accelerates, releasing more Ti ions that stimulate pro-inflammatory cytokine and exacerbate peri-implant inflammation [41].

Whilst assessing the microbial populations on implant surfaces offers an insight into the potential for the surface to harbour or control pathogenic biofilms, it is the impact on immune response that is key to peri-implantitis. Therefore, we designed our model to enable the implant disk-derived biofilms to be directly co-cultured with host epithelial cells. Other models have combined these elements, but in many cases, the biofilms are first cultured on a membrane or glass slide prior to co-culture with implant materials and host cells, rather than grown directly on the implant [42,43]. This could have substantial influences on the enrichment of certain species, as observed in the sequencing data presented here. Furthermore, these existing models test biofilms consisting of distinct species, without assessing the biofilm formation from complex inocula, as we have demonstrated.

By assessing the biofilm cultured directly onto the implant surface, the material itself and host epithelial cells in one holistic, *in vitro* system, our model benefits from the inclusion of any subtle interactions between these elements. Although this does not provide the true complexity of the *in vivo* environment, it allows a greater understanding of the multifactorial influence on peri-implant disease markers.

Given that outer membrane vesicles (OMVs) secreted by bacteria modulate immune responses, particularly those from oral pathogens like *P. gingivalis* [44] and *F. nucleatum* [45], extract experiments were performed to distinguish the effects of cellular and extracellular bacterial components. The immune response indicated that biofilm extracts only accounted for 0–12.5% of total cytokine output, indicating that direct bacterial cell contact drives most of the immune activation. The minimal IL-8 response from extracts supports this, as IL-8 is typically induced by pattern recognition receptors, (e.g. TLR2, TLR4 or NOD) detecting intact bacterial structures [46].

Contact assays, which are more reflective of the interactions in the oral cavity, showed that sandblasted disks induced the strongest immune responses, aligning with their higher biomass and roughness. These findings, in line with cytokine concentrations found in gingival crevicular fluid from peri-implantitis patients [47], emphasise the role of surface design in modulating host responses. Complementary strategies, such as antimicrobial coatings or nano-texturing, may further reduce microbial burden and improve implant outcomes.

Nonetheless, a key limitation of this approach is distinguishing whether reduced cytokine levels were due solely to variations in microbial challenge or also influenced by diminished host cell viability after co-culture. It is possible that greater cytokine differences would have emerged had fewer host cells been compromised during the assays.

Although our model incorporates key *in vivo* elements, such as a salivary pellicle and epithelial co-culture, it remains an approximation. Future improvements could include a constant media flow to better imitate salivary dynamics and using implant abutment configurations to reflect adhesive forces. Cell monolayers do not fully represent host tissue architecture, so organotypic models challenged with implant-derived biofilms may yield more physiologically relevant findings. Furthermore, our model represents a worst-case scenario, allowing uninhibited biofilm maturation without the oral hygiene interventions, which likely produce denser biofilms than typically observed clinically and omits abrasive effects. Nonetheless, by integrating material, microbiological and immunological components, our model provides valuable insight into the complex interactions between implant surfaces and the host environment.

All test materials met ISO 10993-5 biocompatibility standards, as expected given the routine clinical use of CoCr and Ti-6Al-4V. However, incorporating this testing within our model remains valuable, particularly when evaluating novel materials or surface coatings that could alter cellular responses.

## Conclusions

By investigating the inflammatory response to implant disk-derived biofilms, this study contributes to a deeper understanding of how novel implant surfaces may help prevent peri-implantitis. While future work will incorporate three-dimensional organotypic epithelial modelling to better replicate host–microbe interactions, the current findings provide a strong foundation for evaluating potential antimicrobial coatings and surface-finishes. These approaches will support the identification of optimal implant materials and enhance our understanding of host‒surface interactions.

## Supplementary Material

Supplementary material Supplementary Influence of Dental Implant Surfaces on Oral Biofilms and Host Immune Response v3

## Data Availability

Sequence data is available on the Sequence Read Archive (SRA) under accession number: PRJNA1297704.
