# Supplementary material for: Influence of dental implant surfaces on oral biofilms and host immune response
Source: J Oral Microbiol. 2025 Dec 25;18(1):2607199. doi: 10.1080/20002297.2025.2607199 (PMC12777868; doi:10.1080/20002297.2025.2607199)
Supplement: Supplementary material — Supplementary Influence of Dental Implant Surfaces on Oral Biofilms and Host Immune Response v3 [file ZJOM_A_2607199_SM9969.docx]

**Supplementary Materials**

Supplementary Table 1: List of implant materials and surface finishes tested. § Etching performed after high polish.

| Implant Material | Surface Finish |
| --- | --- |
| CoCr | Al_2_0_3_ Sandblasted |
|  | High Polish |
|  | Electrolytic Polish |
| Ti-6Al-4V | Al_2_0_3_ Sandblasted |
|  | High Polish |
|  | HCl etched ^§^ |
|  | H_2_SO_4_ etched ^§^ |
|  | HNO_3_ etched ^§^ |
| Hydroxyapatite | Control |

**Sterilised human saliva**

Human saliva from a healthy subject was mixed with dithiothreitol (DTT) to a final concentration of 2.5mM, gently agitated for 10 minutes and centrifuged at 4,000 rpm, 4°C for 20 minutes. The supernatant was diluted 50:50 with phosphate buffered saline (PBS) and syringe filter sterilised (0.22 µm). Sterility was assessed by plating 5 µL on to blood agar incubated both anaerobically and in 5% CO^2^ for 72 hours.

**Artificial saliva constituents**

75% artificial saliva (porcine gastric mucin 10 g L^-1^, NaCl 0.381 g L^-1^, KCl 1.114 g L^-1^, ascorbic acid 0.002 g L^-1^, urea 0.541 g L^-1^, arginine 0.871 g L^-1^), 25% basal medium (proteose peptone 10 g L^-1^, trypose peptone 5 g L^-1^, yeast extract 5 g L^-1^, L-cysteine hydrochloride 0.5 g L^-1^, haemin 0.0002 g L^-1^, menadione 0.00004 g L^-1^).

Supplementary Table 2: Normalised mass percentages of acid-etched Ti-6Al-4V disks by energy dispersive spectroscopy.

|  | Mass Norm. (%) | | | | |  |
| --- | --- | --- | --- | --- | --- | --- |
|  | Aluminium | Vanadium | Titanium | Carbon | Sulphur | Chlorine |
| HCL | 5.499 | 4.050 | 86.278 | 4.173 | - | 0.0001 |
| Nitric Acid | 5.298 | 3.235 | 83.782 | 3.546 | 1.285 | - |
| Sulphuric Acid | 5.268 | 3.366 | 83.260 | 4.635 | 1.994 | - |


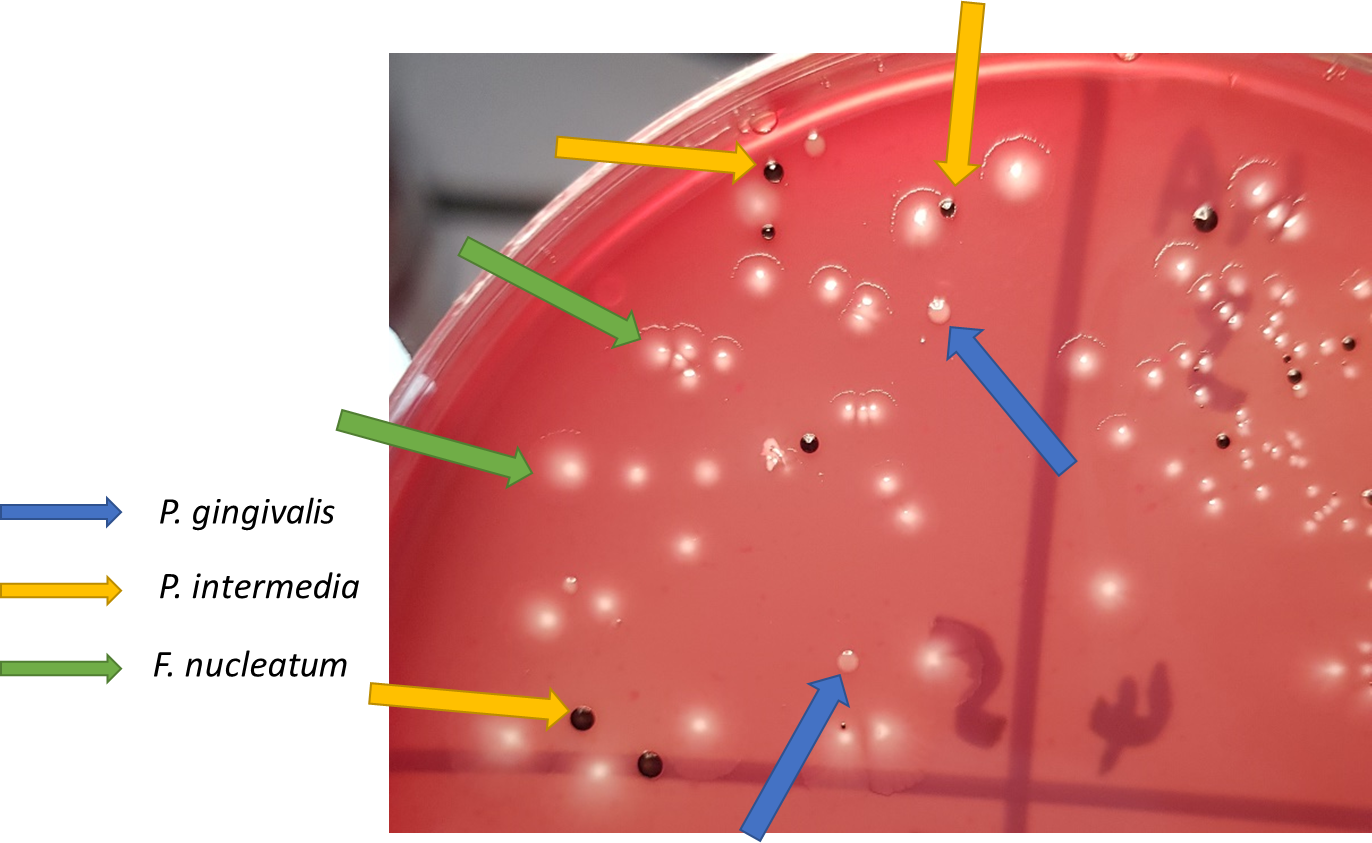


Supplementary Figure 1: Photographic representation of colony differentiation on Columbia blood agar (CBA) supplemented with 75 mg L^-1^ vancomycin, incubated anaerobically for 5 days. Black pigmented colonies (yellow arrows) are Prevotella intermedia, colonies with feather edges (green arrows) are Fusobacterium nucleatum, and round grey colonies (blue arrows) are Porphyromonas gingivalis. If left for 2 more days, the P. gingivalis colonies will start to pigment and become indistinguishable from P. intermedia.

**
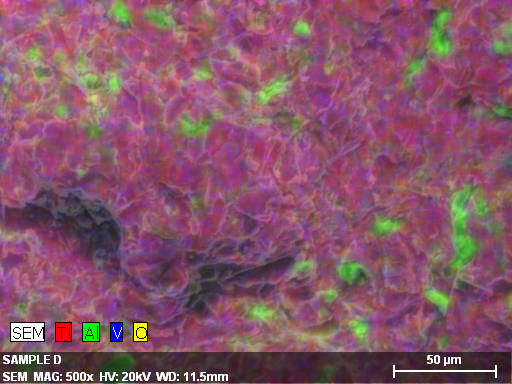
**

Supplementary Figure 2: Visual representation of elemental mass mapping on the surface of a Al_2_O_3_ sandblasted Ti-6Al-4V. Image taken at 500x magnification at 20kV using a Hitachi S-3400N in secondary electron mode and a 2x 60mm2 Flash6 energy dispersive spectrometry system.


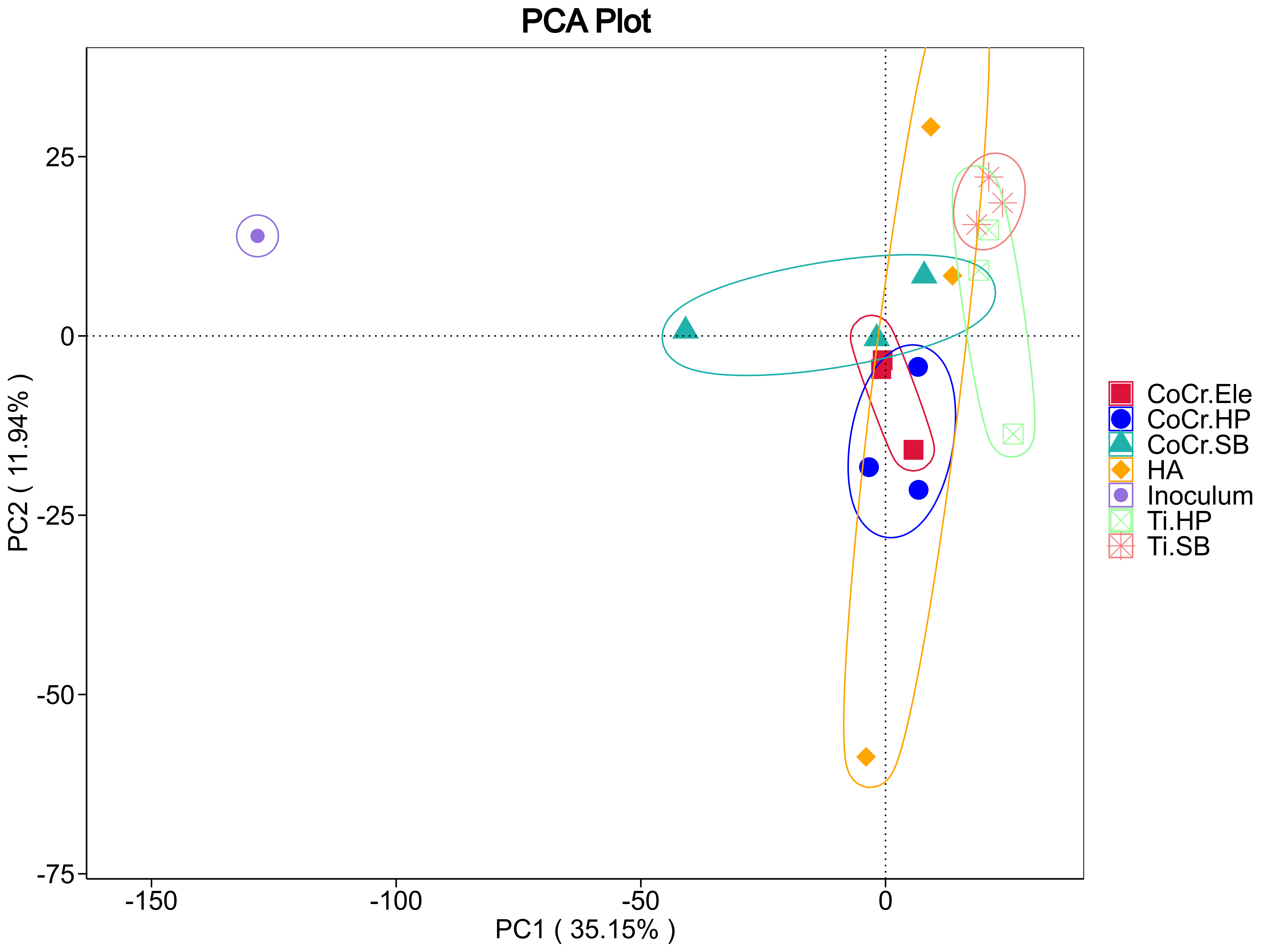


Supplementary Figure 3: PCA plot demonstrating the functional prediction analysis of biofilm populations cultured on different implant surfaces and the inoculum using PICRUSt2 and Clusters of Orthologous Groups database.

**


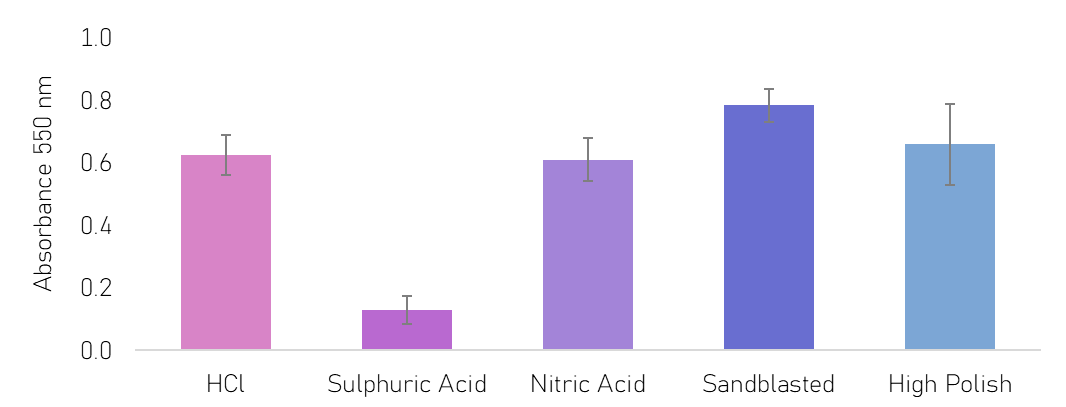

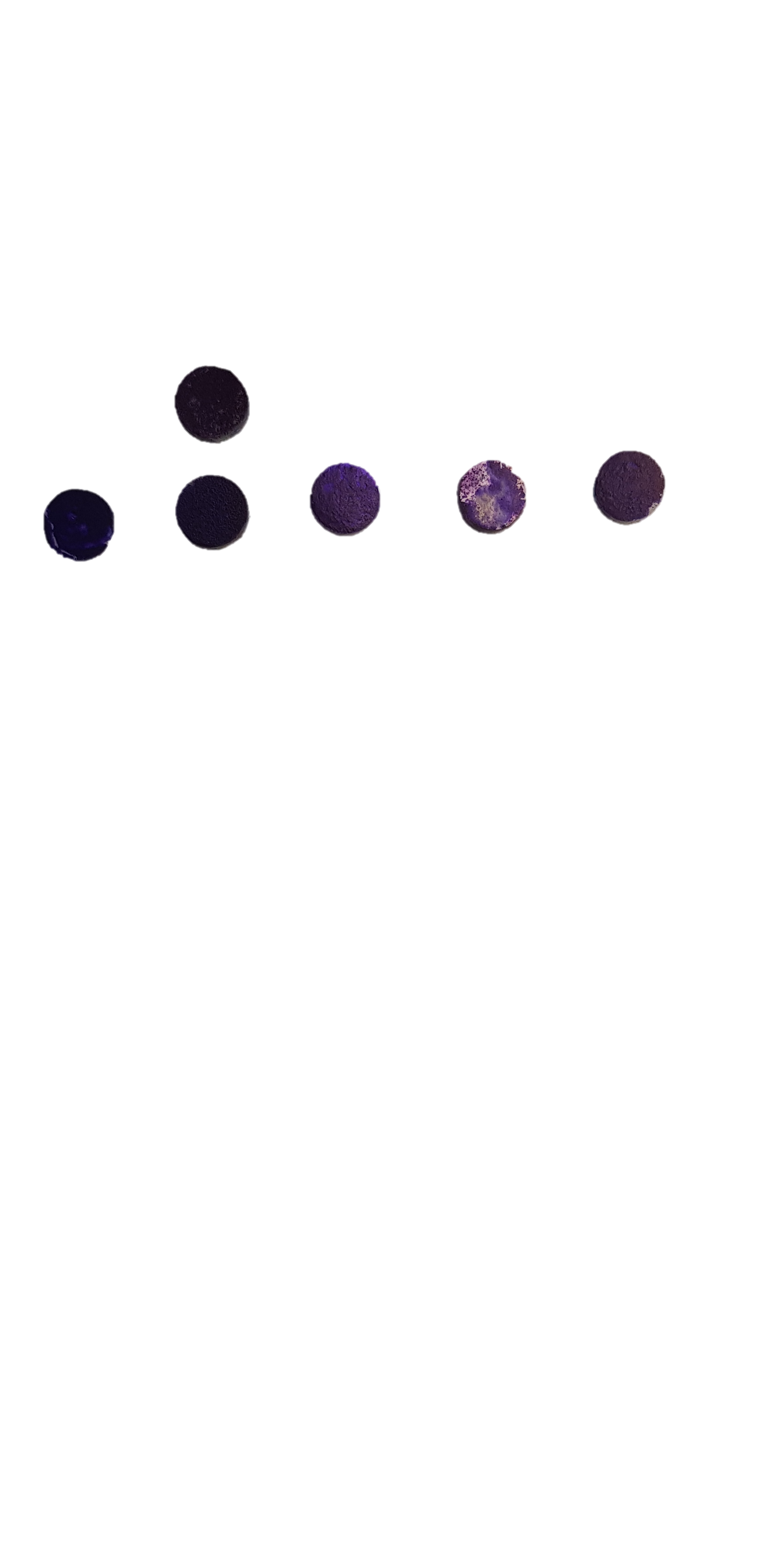

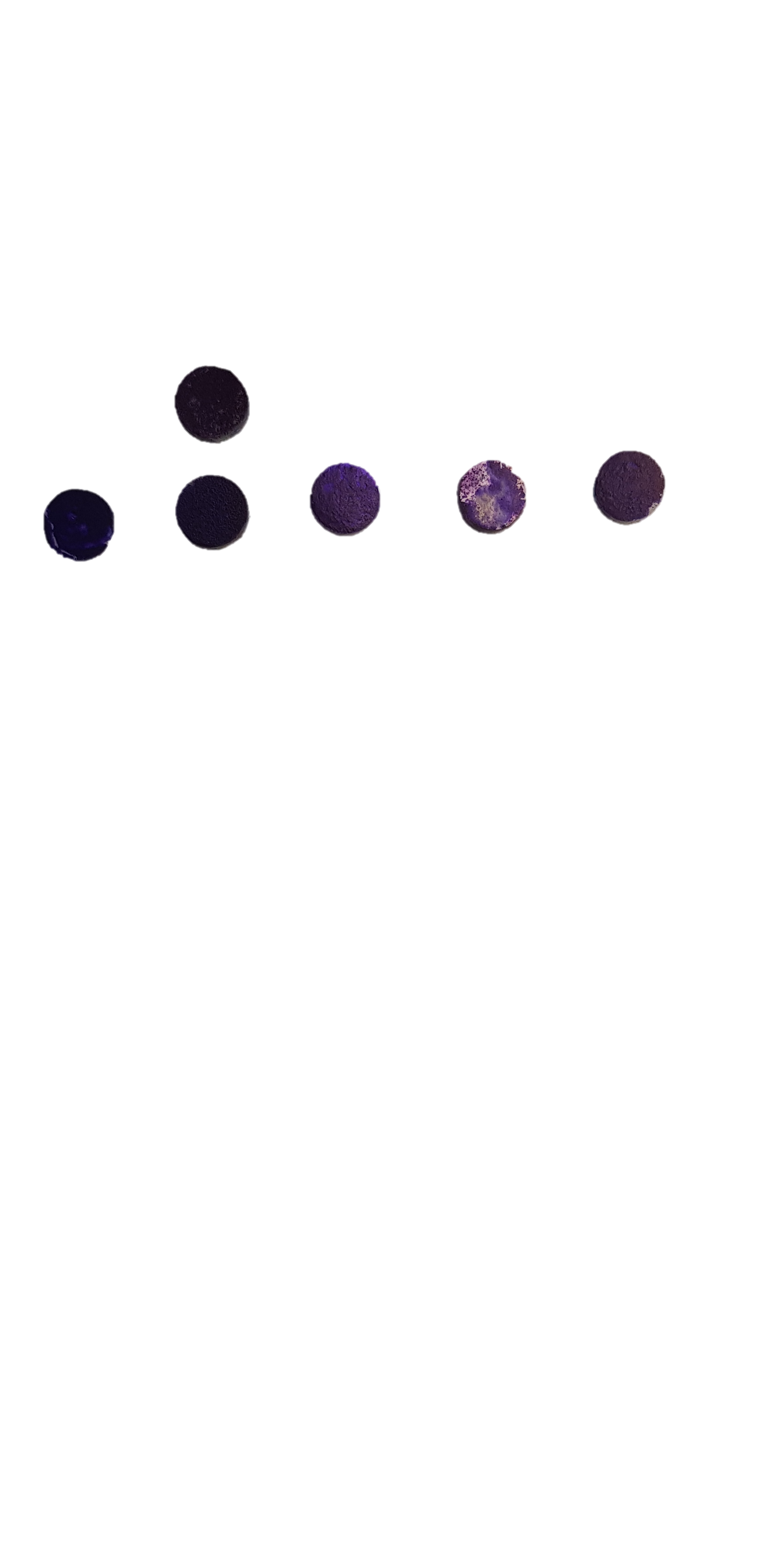

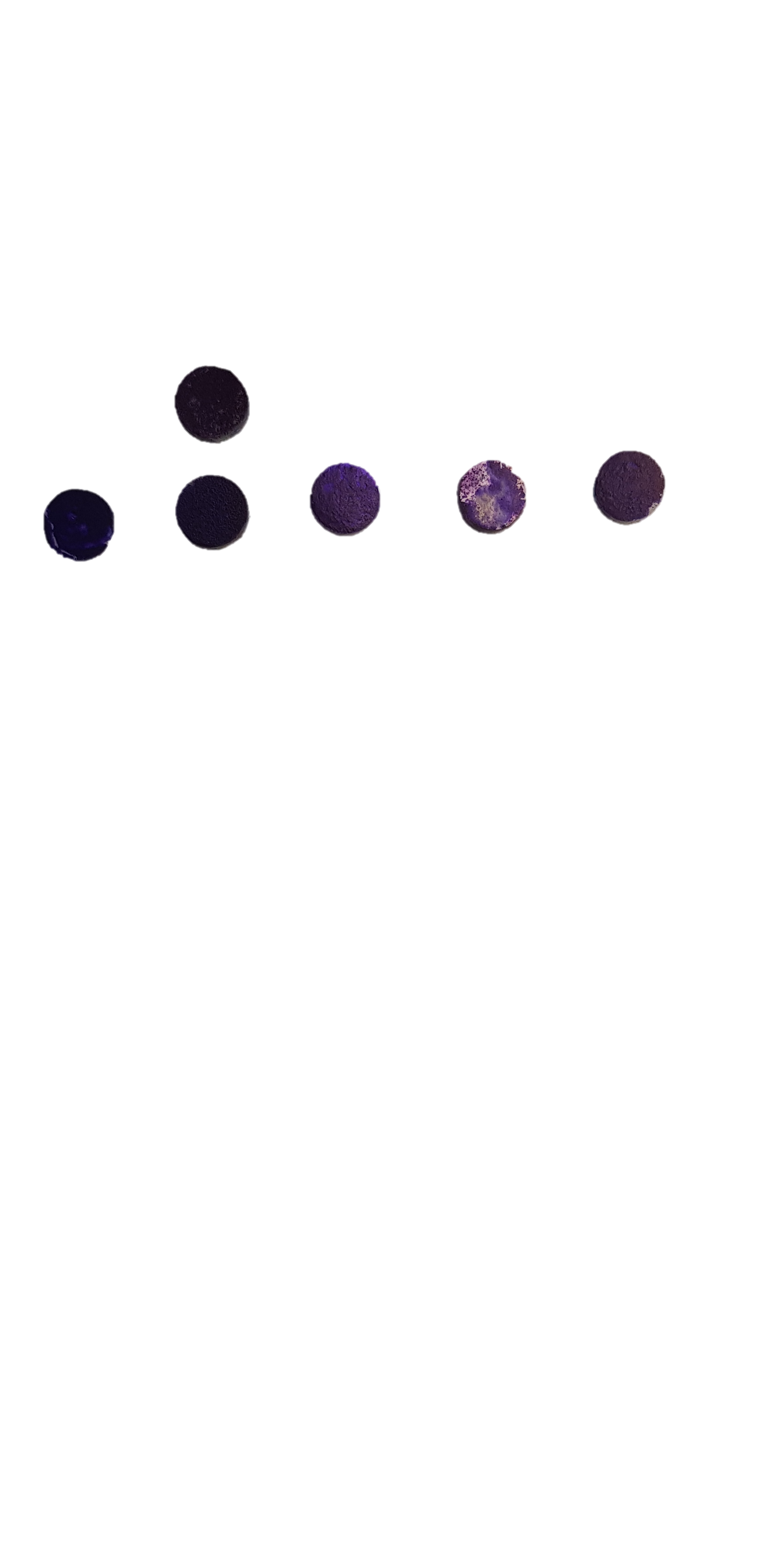

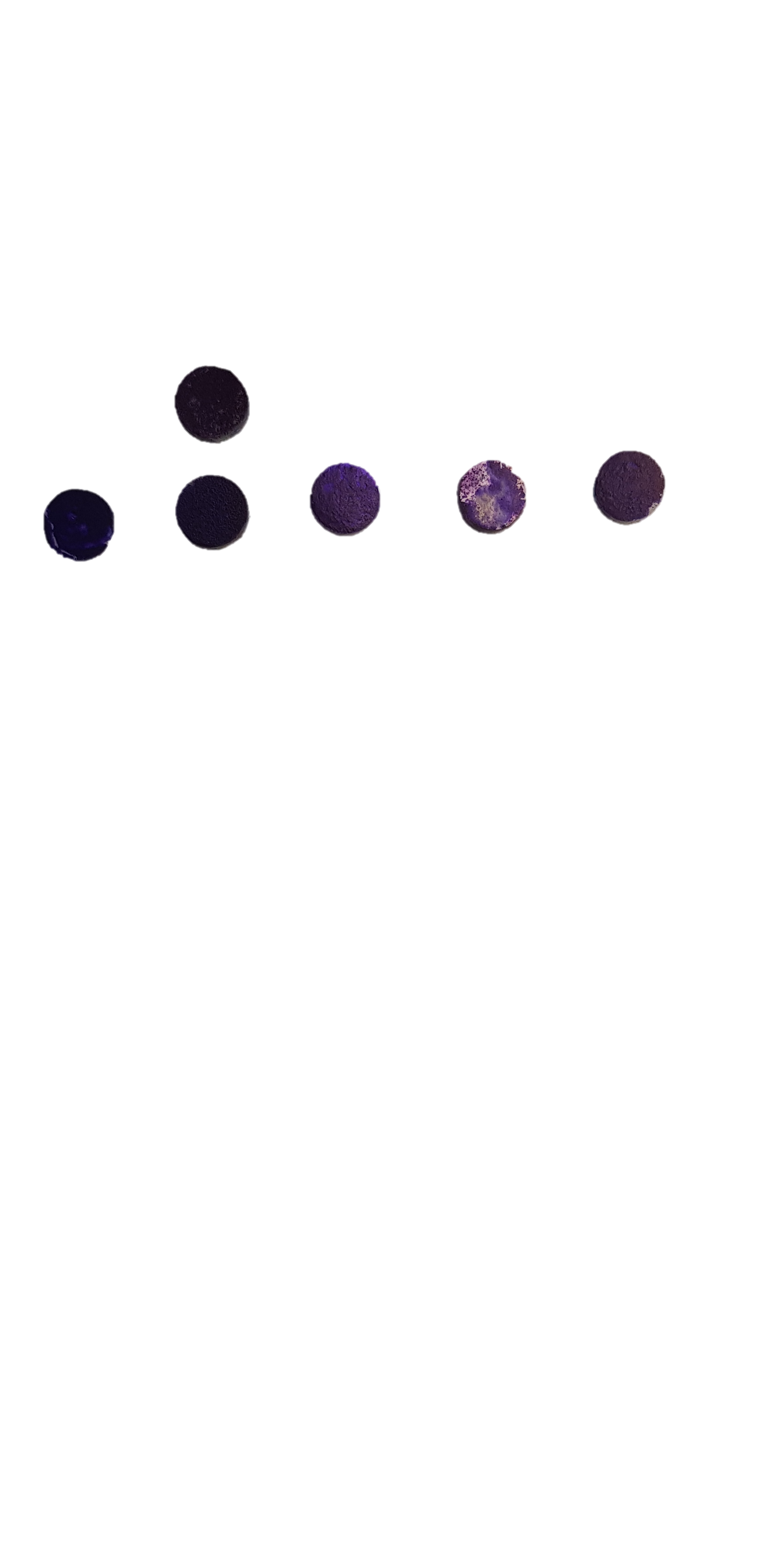

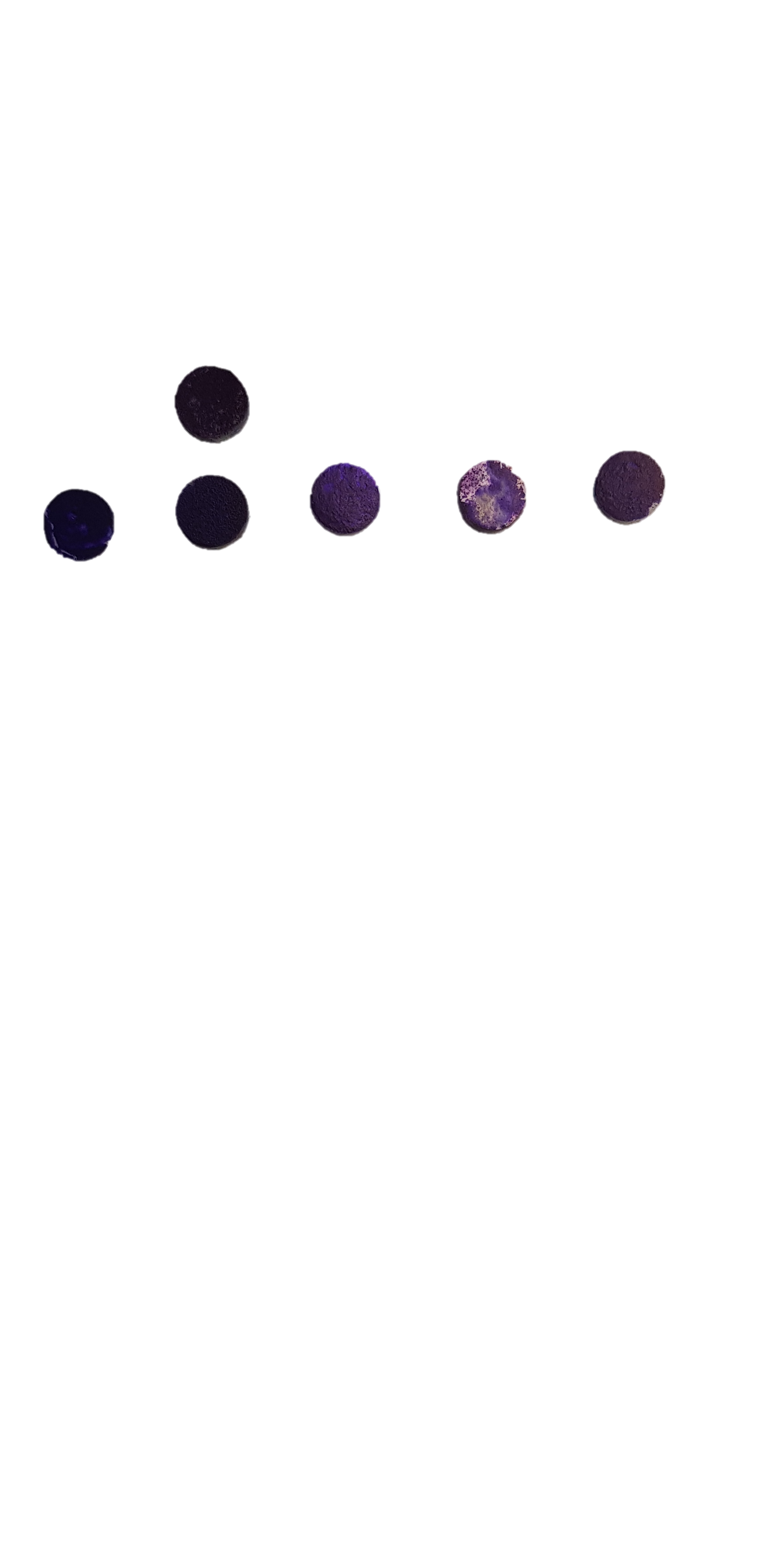


Supplementary Figure 4: Biofilm biomass of mature complex oral biofilms on Ti-6Al-4V disks with different acid-etched surfaces determined by crystal violet assay. Standard error of the mean is shown. Top – photographic record of crystal violet stained biofilms. Statistical comparison by ANOVA ** p<0.001

Supplementary Figure 5: Dysplastic oral keratinocyte (D.O.K.) monolayer viability post 24 hr co-culture with direct contact and biofilm extract, determined by MTT assay. Standard error of the mean is shown.

*
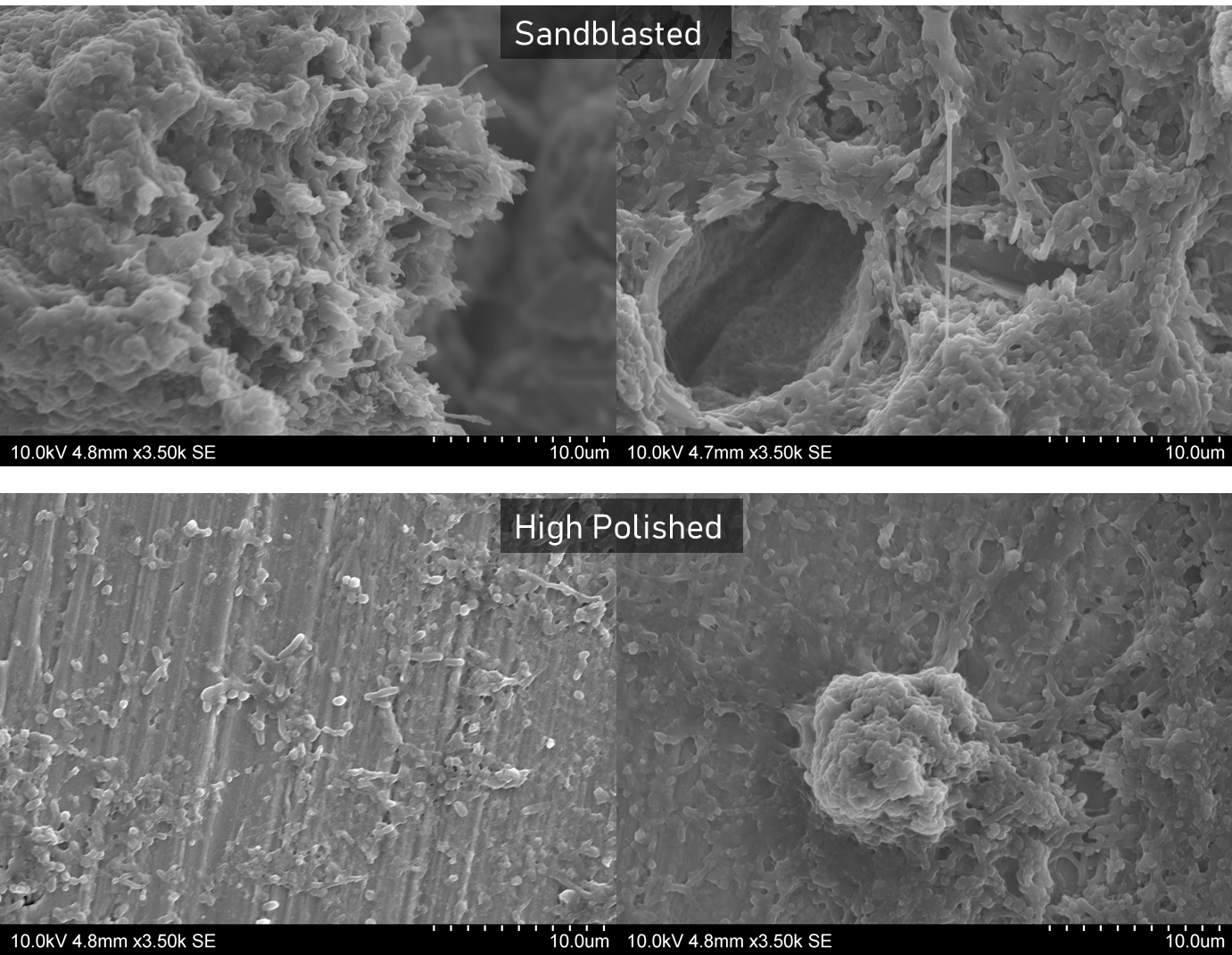
*

*Supplementary Figure 6: Scanning electron microscopy images complex oral biofilms cultured on sandblasted (top) and high polished (bottom) titanium surfaces at 3,500 x magnification (10kV).*
